# Stem cells from human exfoliated deciduous teeth correct the immune imbalance of allergic rhinitis via Treg cells in vivo and in vitro

**DOI:** 10.1186/s13287-019-1134-z

**Published:** 2019-01-22

**Authors:** Yu-Yang Dai, Si-Yang Ni, Ke Ma, Yu-Shi Ma, Zhi-Shi Wang, Xiu-Li Zhao

**Affiliations:** 1 National Institute for Drug Clinical Trial, Beijing Tongren Hospital, Capital Medical University, 1 Dongjiaominxiang Road, Beijing, 100730 China; 2 College of Chemical Biology and Pharmaceutical Sciences, Capital Medical University, 10 Xitoutiao Road, Beijing, 100069 China; 3 Beijing Institute of Ophthalmology, Beijing Tongren Eye Center, Beijing Tongren Hospital, Capital Medical University, 9 Chongwenmennei Road, Beijing, 100005 China; 4 Beijing Tason Biotech Co. Ltd., 10 PKUCare Industrial Park, Beijing, 102206 China

**Keywords:** Allergic rhinitis, Stem cells from human exfoliated deciduous teeth, Regulatory T cells, Transforming growth factor beta, Immunoregulation

## Abstract

**Background:**

Several studies have demonstrated that mesenchymal stem cells can ameliorate the inflammation of allergic rhinitis (AR) and correct the Th1/Th2 immune imbalance.

**Methods:**

This study was performed to explore the immunomodulation properties of stem cells from human exfoliated deciduous teeth (SHEDs) in the treatment of AR in vivo and in vitro. BALB/c mice were sensitized to ovalbumin (OVA) by intraperitoneal injection, and then SHEDs or bone marrow mesenchymal stem cells (BMMSCs) were injected intravenously before challenge. We evaluated nasal symptoms, inflammatory infiltration of nasal mucosa, immunoglobulin secretion, cytokine production, and mRNA expression in the spleen. In addition, peripheral blood mononuclear cells (PBMCs) from AR patients were cultured with SHEDs or BMMSCs in the presence of phytohemagglutinin (PHA). PBMCs cultured alone with or without PHA served as controls. After 3 days of culture, we examined the effect of SHEDs on T lymphocyte proliferation, cytokine secretion, and the proportion of Foxp3^+^ Treg cells via flow cytometry. Finally, to determine the role of soluble factors (TGF-β_1_, PGE_2_) in the immunomodulatory mechanism, a cytokine neutralization assay was performed.

**Results:**

Nasal symptoms and inflammatory infiltration were significantly reduced after SHED administration. The OVA-specific IgE and IgG_1_ levels in serum were significantly decreased, and the increased IL-4, IL-5, IL-13, and IL-17A levels in the spleen after OVA challenge were markedly downregulated, while the level of IFN-γ was upregulated by SHED administration. The mRNA expression levels also changed correspondingly. SHEDs significantly inhibited the proliferation of T lymphocytes; increased the levels of IFN-γ, IL-10, PGE_2_, and TGF-β_1_; decreased the levels of IL-4 and IL-17A; and induced the expansion of Treg cells in the coculture system. The neutralization of TGF-β_1_ partly relieved the immunosuppression of SHEDs, but blocking PGE_2_ did not. In addition, SHEDs were superior to BMMSCs in inhibiting the Th2 immune response in vivo and inducing the expansion of Treg cells in vitro.

**Conclusion:**

These results suggest that SHEDs could correct the CD4^+^ T cell immune imbalance via Treg cells and may be potential therapeutic agents for the treatment of allergic diseases, such as AR, in the future.

**Electronic supplementary material:**

The online version of this article (10.1186/s13287-019-1134-z) contains supplementary material, which is available to authorized users.

## Background

Allergic rhinitis (AR) is an allergic inflammatory reaction characterized by sneezing, itching, rhinorrhea, and nasal blockage. The incidence of AR has increased in recent years, and it seriously impacts the quality of life and work efficiency of patients [1]. Pharmacotherapy, such as intranasal corticosteroids, antihistamines, and leukotriene antagonists, can reduce allergic symptoms, and allergen immunotherapy can be employed if patients are resistant to the usual therapy. Immunotherapy involves a regular injection of incremental doses of allergen vaccines to induce allergen tolerance. However, immunotherapy is restricted by allergen types, and the long treatment cycle usually results in poor compliance in clinical settings.

In recent years, experimental and clinical evidence have indicated that mesenchymal stem cells (MSCs) possess significant immunomodulatory and nonimmunogenic properties in the treatment of several immune diseases, including asthma, multiple sclerosis, Crohn’s disease, graft-versus-host disease, and other inflammatory disorders [2–4]. MSCs have been proposed as a new approach for AR treatment as they are able to reduce eosinophil infiltration in the nasal mucosa and regulate the release of cytokines to control the CD4^+^ T cell response [5–7]. However, the main problem at present is to find the suitable tissue source from which MSCs can be achieved by immune regulation, security, easy access, low cost, and no ethical hurdles.

MSCs were first found in the bone marrow, namely bone marrow mesenchymal stem cells (BMMSCs). The immunomodulatory ability of BMMSCs has been verified in various allergic disease models with typicality and representativeness [8]. Nevertheless, the acquisition of BMMSCs is difficult along with invasive operations. The content of MSCs in bone marrow is relatively low, and the quantity and differentiation capacity reasonably decrease with age [9]. Fortunately, after the first isolation of MSCs from bone marrow, further studies demonstrated that other tissues also contain MSCs. To date, MSCs can be obtained more easily from many tissues, such as adipose tissue, umbilical cord, kidney, lymph nodes, and dental pulp. Among them, stem cells from human exfoliated deciduous teeth (SHEDs) are drawing attention. SHEDs were first isolated from children’s deciduous teeth [10], which are regarded as medical waste. Compared with BMMSCs, they are easily obtained and are free from the ethical concerns. The dental pulp from deciduous teeth exists before birth and until permanent teeth eruption, SHEDs are considered more immature than other postnatal MSCs. Besides all, SHEDs are MSCs with a more proliferative capacity than BMMSCs [11]. Up to now, several SHED banks have been established worldwide. SHEDs are relatively new and less studied than MSCs from other sources. Few studies have investigated the immunomodulatory properties of SHEDs in AR models. In this study, we addressed the immunomodulatory effects of SHEDs on a murine model of AR and a cell coculture system, providing a basis for further clinical applications of MSCs in treating allergic diseases.

## Methods

### Expansion of SHED and BMMSC in vitro

Three frozen tubes of SHEDs (passage 2) were kindly provided from Tason Biotech Co., Ltd. (Beijing, China) for this research study. These cells had been isolated from the pulp tissues of exfoliated deciduous teeth in children aged 6–8 years. The immunophenotyping (CD11b^−^/CD19^−^/CD34^−^/CD45^−^/CD73^+^/CD90^+^/CD105^+^) and capacity of differentiation (osteoblast induction) were confirmed by the supplier. SHEDs were seeded into T75 flasks, cultured in complete medium containing alpha modification of Eagle’s medium (α-MEM; Invitrogen, Carlsbad, CA, USA) supplemented with 15% fetal bovine serum (FBS; Invitrogen), 2 mM L-glutamine (Invitrogen), 100 μM l-ascorbic acid 2-phosphate (Sigma, St. Louis, MO, USA), and 1% penicillin/streptomycin (Invitrogen) for further passages. The culture medium was changed every 3 to 4 days, and the cells were again trypsinized and cultured for additional passages when they reached 80% confluency. When SHEDs were cultured to passage 4, 15% FBS was replaced with 10% FBS. These steps were repeated until a sufficient number of SHEDs (passage 6) were collected for further experiments.

BMMSCs were purchased as a T25 cell culture flask (passage 2) from Lonza (Basel, Switzerland). To obtain the required number of cells, BMMSCs were cultured in complete Dulbecco’s modified Eagle’s medium/nutrient F-12 Ham (DMEM/F12; Invitrogen) supplemented with 10% FBS, 2 mM l-glutamine, and 1% penicillin/streptomycin, and cells were also expanded in vitro as SHEDs.

### AR mouse model

Female BALB/c mice (6–8 weeks of age), free of murine-specific pathogens, were purchased from Vital River Laboratory Animal Technology Co., Ltd. (Beijing, China). The mice were housed in a controlled environment under a 12/12-h light/dark cycle with free access to food and water. The animal study protocol was approved by the Institutional Animal Care and Use Committee of Capital Medical University (AEEI-2018-075).

**Fig. 1:**
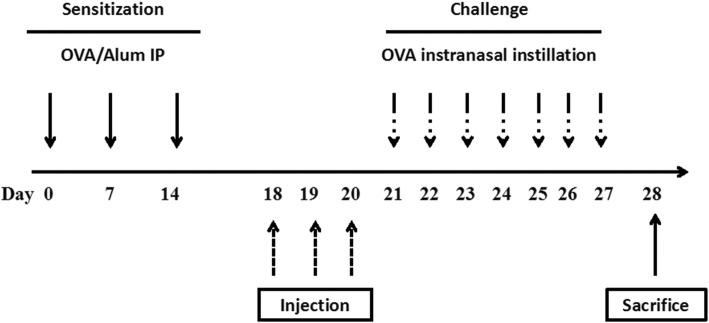
The animal experimental protocol. Mice were sensitized on days 0, 7, and 14 by an intraperitoneal injection of OVA with aluminum hydroxide. From days 21 to 27, the mice were challenged with OVA intranasal instillation using a micropipette. Purified SHEDs or BMMSCs were injected via the tail vein on days 18, 19, and 20, and all mice were sacrificed on day 28

AR mouse models were sensitized using ovalbumin (OVA, grade V; Sigma) and alum (Pierce, Rockford, IL, USA) as previously reported with minor modification [12]. Briefly, mice were sensitized on days 0, 7, and 14 via intraperitoneal injection of 25 μg OVA emulsified in 2 mg alum in a total volume of 100 μl phosphate-buffered saline (PBS). On days 21 to 27 after the initial sensitization, these mice were challenged by the daily intranasal instillation of 100 μg OVA in 20 μl PBS using a micropipette. The mice were sacrificed via cervical dislocation under anesthesia at day 28 (Fig. 1).

### Xenograft of SHEDs or BMMSCs into AR mouse model

SHEDs or BMMSCs (passage 4) were washed and suspended in PBS at a concentration of 5 × 10^6^ cells/ml, and 100 μl of purified stem cells was injected with a 26-gage needle into the mouse tail vein on days 18, 19, and 20 (Fig. 1). Thirty mice were divided into five groups of six mice, each in accordance with the different interventions of sensitization, challenge, and injection as follows: (1) control group, sensitized and challenged with PBS and injected with PBS; (2) sham-SHED group, sensitized and challenged with PBS and injected with SHEDs; (3) OVA group, sensitized and challenged with OVA and injected with PBS; (4) SHED group, sensitized and challenged with OVA and injected with SHEDs; (5) BMMSC group, sensitized and challenged with OVA and injected with BMMSCs.

### Nasal allergic symptoms

On day 27, during the 10-min period after the final administration of intranasal OVA, the frequencies of sneezing and nasal rubbing were recorded by two observers in a blind manner as previously reported [13].

### Histologic analysis and immunohistochemistry

The mice were sacrificed 24 h after the last OVA challenge. Nasal mucosa was removed, washed with PBS, and immersed in 4% buffered paraformaldehyde for 24 h. After fixation, the specimens were embedded in paraffin wax and sectioned serially at a thickness of 4 μm. Sections were stained with hematoxylin and eosin (HE) and periodic acid-Schiff (PAS) respectively and observed under a light microscope (× 400 magnification). HE staining was performed to evaluated eosinophil infiltration. Eosinophils were morphologically defined by the presence of eosinophilic granules (red) that were stained by eosin in the cytoplasm and the presence of a two-lobed nucleus. The numbers of eosinophils were evaluated in four randomly selected fields by two observers blinded to the experiment. PAS staining was used to estimate goblet cell hyperplasia and mucus production. Positive-goblet cells were stained purple-magenta color under the microscope.

To analyze T lymphocyte infiltration, immunohistochemistry (IHC) procedure was performed using Elivision method. Briefly, after deparaffinization and rehydration, endogenous peroxidase activity was quenched with 3% hydrogen peroxide. Sections were heated for 3 min in a citrate buffer (10 mmol/L, pH 6.0) to retrieve antigen. After goat serum blocking for 20 min, sections were incubated with the primary polyclonal antibody, rabbit anti-mouse CD3e (1:200; Cat.No. PA5-32318; Invitrogen), overnight at 4 °C to stain target protein expression. Following 20-min incubation with secondary anti-rabbit antibody, 3.3-diaminobenzidine (Sigma) was applied for visualizing immunoreactivity, and counterstaining was performed with Mayer hematoxylin. The cytoplasm of the positive T lymphocytes was stained brown.

### Detection of serum OVA-specific IgE, IgG_1_, and IgG_2a_

The blood samples were collected from mice via eyeball extraction 24 h after the last OVA challenge. Serum levels of OVA-specific IgE, IgG_1_, and IgG_2a_ were measured by ELISA kits (BD Bioscience, San Jose, CA, USA) in accordance with the manufacturer’s instructions.

### Expression of cytokines in the spleen

Fresh spleens were separated from the mice in aseptic conditions after sacrifice. The spleen was placed in a petri dish containing RPMI 1640 medium, and a syringe was used to crush the tissue before filtration through a 70-μm filter. The splenocytes were treated with lysis buffer to deplete erythrocytes. Single-cell suspensions of splenocytes (1 × 10^5^ cells/well in 96-well culture plates) were cultured in completed RPMI 1640 medium supplemented with 10% FBS, 2 mM l-glutamine, and restimulated with OVA (100 ng/ml). After 3 days of culture, interleukin (IL)-4, IL-5, IL-13, and IL-17A and interferon (IFN)-γ levels in the culture supernatant were measured using an ELISA kit (BD Bioscience) following the manufacturer’s instructions.

### Quantitative real-time polymerase chain reaction analysis

Total RNA was extracted from the spleen using the TRIzol reagent kit (Invitrogen) for messenger RNA (mRNA) relative expression analysis of IFN-γ, T-bet, IL-4, GATA-3, IL-17A, RORγt, and Foxp3. Equivalent amounts of RNA were reverse transcribed using the PrimeScript RT Reagent Kit (Takara, Tokyo, Japan). The mRNA expression analysis was performed using an Applied Biosystem 7500 Real-Time PCR System (Applied Biosystems, Foster City, CA, USA). The corresponding primers and probes for the cytokines and chemokines are listed in Additional file 1: Table S1. The average transcript levels of genes were normalized to β-actin. The relative mRNA gene expression was calculated using the 2^-∆∆Ct^ method.

### Coculture of SHEDs with PBMCs

To investigate the underlying mechanism of the SHED immunomodulatory effect, we carried out coculture experiments of MSCs with human peripheral blood mononuclear cells (PBMCs). PBMCs were isolated from heparinized peripheral blood of 20 AR patients using Ficoll-Paque (Amersham Biosciences, NJ, USA). The diagnosis of seasonal AR was established according to the criteria of the Allergic Rhinitis and its Impact on Asthma (ARIA) [14] based on clinical symptoms, nasal endoscopy examination, and serum IgE allergen detection. The patients did not receive intranasal steroids, antihistamines, or leukotriene blocker treatments within 1 week or oral steroids within 3 months prior to this study. The Ethics Committee of Beijing Tongren Hospital, Capital Medical University approved this study (TREC2018-KY01), and informed consent was obtained from all participants.

Coculture experiments (*n* = 10) were performed in a 24-well plate, and both types of human MSCs were treated with mitomycin (incubation for 2 h at 10 μg/ml) to prevent proliferation in advance. Next, 1 × 10^5^/well SHEDs or BMMSCs (passage 6) were cultured in complete RPMI 1640 culture medium (Invitrogen) supplemented with 10% FBS, 2 mM glutamine, 100 U/ml penicillin, and streptomycin, and allowed to adhere for 2 h at 37 °C and 5% CO_2_. Then, 1 × 10^6^ PBMCs were seeded onto the cultured MSCs in the presence of 5 μg/ml phytohemagglutinin (PHA; Sigma). PBMCs cultured alone with or without PHA served as controls. After 3 days, the suspended PBMCs were collected for flow cytometry analysis. The supernatant of cell cultures was collected and kept at − 80 °C for later examination with ELISA.

### Lymphocyte proliferation assay

The effects of SHEDs and BMMSCs on lymphocyte proliferation were examined using carboxyfluorescein diacetate succinimidyl ester (CFSE; BD Biosciences) labeling (*n* = 5). Briefly, PBMCs were resuspended and labeled with CFSE (5 μM) for 10 min at 37 °C and 5% CO_2_. Then, 1 × 10^6^ CFSE-labeled PBMCs were then seeded into a set of wells containing diminishing numbers of MSCs (1 × 10^4^/well SHEDs, 2 × 10^4^/well SHEDs, 1 × 10^5^/well SHEDs, and 1 × 10^5^/well BMMSCs) in the presence of 5 μg/ml PHA. CFSE-labeled PBMCs cultured alone with or without PHA served as controls. After 3 days of culture, gated CD3^+^ T cells in PBMCs were analyzed using a FACS Calibur flow cytofluorimeter (BD Biosciences). The percentages of mean fluorescent intensity (MFI) were used to reflect the proliferation of T lymphocytes.

### Cytokine measurement assay

The concentrations of human IFN-γ, IL-4, IL-10, IL-17A, prostaglandin-E2 (PGE_2_), and transforming growth factor beta 1 (TGF-β_1_) in the supernatant of the coculture systems were examined using ELISA kits (IFN-γ, IL-4, IL-10, IL-17A: BD Biosciences; PGE_2_: R&D System, Minneapolis, MN, USA; TGF-β_1_: Thermo Fisher, Waltham, MA, USA) in accordance with the manufacturer’s instructions.

### Flow cytometry analysis

Flow cytometry analysis was performed to identify Treg cells. Cells were stained with fluorescein isothiocyanate (FITC)-labeled anti-CD4 antibodies (Abs) and allophycocyanin (APC)-labeled anti-CD25 Abs (BD Biosciences) and then fixed and permeabilized using a fix/perm solution (BD Biosciences) according to the manufacturer’s instructions. The cells were then incubated with phycoerythrin (PE)-labeled anti-Foxp3 Abs (BD Biosciences), and an isotype-matched control was used to determine the background. The data were evaluated using FlowJo software (version 7.6; Treestar, Inc., St Ashland, OR, USA).

### Cytokine neutralization assay

To identify the possible pathway involved in the immune regulation of SHEDs on T lymphocytes, coculture systems of PBMCs and SHEDs were treated with indomethacin (10 μM; Sigma) to block the PGE_2_ pathway through the inhibition of cyclooxygenase (COX) or anti-TGF-β Ab (BD Biosciences), respectively, for the neutralization of soluble TGF-β_1_. Treg expansion was detected via flow cytometry, and T lymphocyte proliferation was evaluated by CFSE labeling (*n* = 5).

### Statistical analysis

Data are represented as the mean ± SD from three independent experiments. Statistical significance was assessed by Student’s *t* test or ANOVA using SPSS software 23.0 (SPSS Inc., Chicago, IL, USA) and GraphPad Prism 7.0 (GraphPad, San Diego, CA, USA). A *p* value < 0.05 was considered significant.

## Results

### SHEDs reduce nasal inflammation in an AR mouse model

**Fig. 2:**
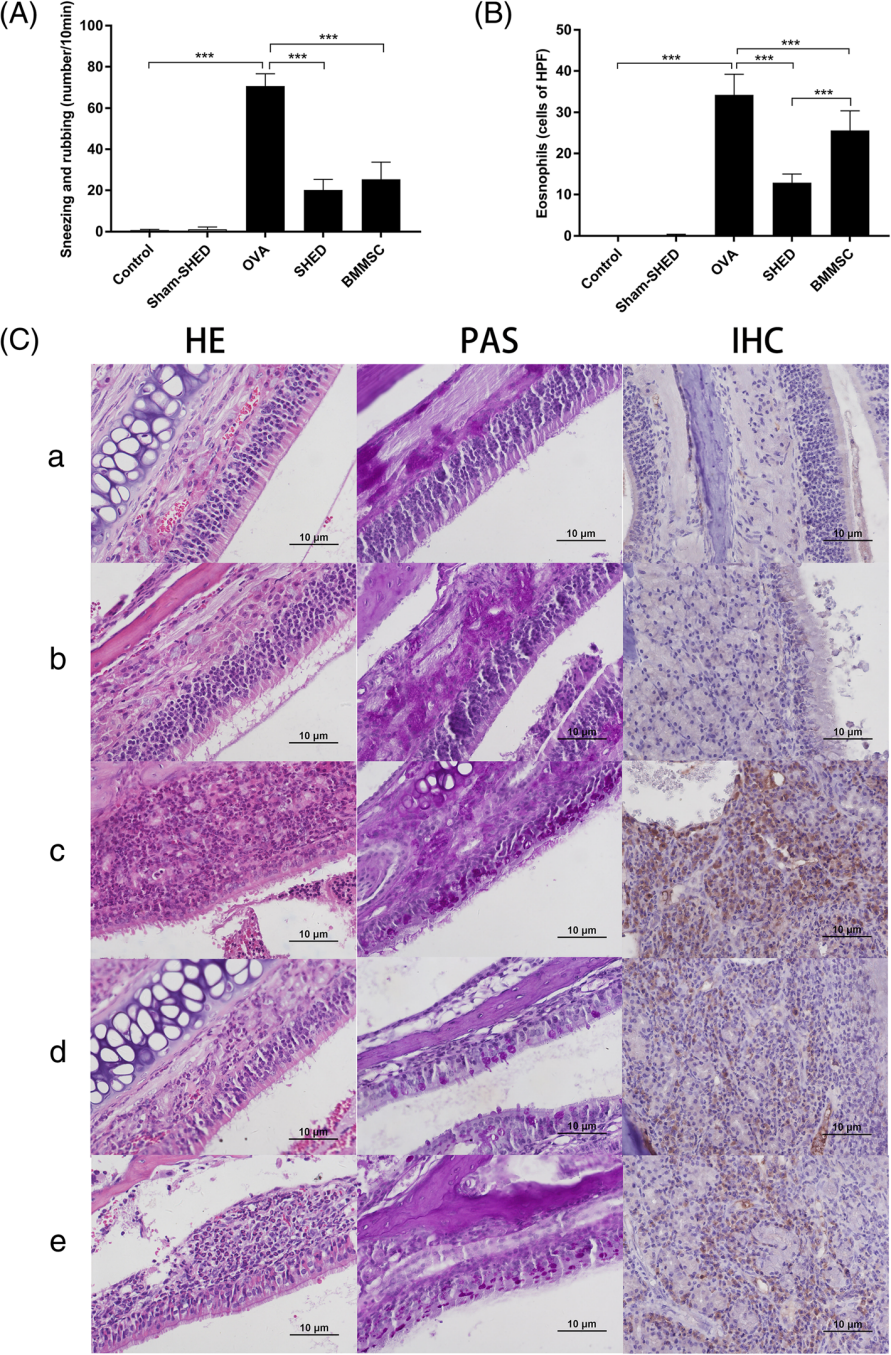
Effect of SHED on nasal inflammation. **A** The nasal symptoms were evaluated by the number of sneezing and rubbing events in 10 min after the final challenge. Nasal symptoms in the OVA group were much more serious than those in the control group and sham-SHED group but were relieved after SHED and BMMSC treatment. **B**, **C** Twenty-four hours after the last challenge, HE staining, PAS staining, and IHC were used to reflect the inflammatory infiltration in the nasal mucosa. The numbers of eosinophils were evaluated under a light microscope (× 400 magnification). Nearly no inflammatory cells were observed in the control group (a) and sham-SHED group (b). In contrast, the OVA group (c) exhibited obvious eosinophil infiltration, goblet cell hyperplasia, and T lymphocyte infiltration. The inflammatory infiltration in the SHED group (d) and BMMSC group (e) was significantly alleviated compared with the OVA group, and the eosinophil count in the SHED group was lower than that of the BMMSC group. Data are expressed as the mean ± SD (*n* = 6 in each group) from three representative experiments. ***p < .001

To assess the anti-inflammatory effects of SHEDs on allergic symptoms, we injected SHEDs into an AR mouse model via the tail vein. First, we counted the sum of sneezing and nasal rubbing events in 10 min after the final challenge. As illustrated in Fig. 2A, the number of sneezing and rubbing events was significantly higher in the OVA group than in the control group and sham-SHED group (*p* < .001). The nasal symptoms of mice were obviously relieved after the injection of MSCs. There was no significant difference between the BMMSC group and the SHED group at the same injection dose.

We qualitatively and quantitatively evaluated the effect of SHED treatment on the histopathology of the nasal mucosa (Fig. 2B, C). The pathological manifestations of AR mainly include eosinophil and T lymphocyte infiltration. T lymphocytes, especially Th2 cells, can release cytokines and recruit inflammatory cells. Eosinophils can release cellular content, cause tissue damage, and promote inflammation progress. According to our results, nearly no inflammatory cell was observed in the nasal mucosa of the control and sham-SHED groups. In contrast, the OVA group exhibited obvious eosinophil infiltration, goblet cell hyperplasia, and T lymphocyte infiltration in the nasal mucosa. Interestingly, the numbers of eosinophils were significantly decreased in the SHED and BMMSC groups compared to the OVA group (both *p* < .001). Meanwhile, the positive-goblet cell and T lymphocyte infiltration were significantly alleviated upon MSC administration. It demonstrated that SHEDs have the ability to inhibit the development of allergic inflammation. In addition, the inflammatory reaction in the SHED group was much lighter than that in the BMMSC group (*p* < .001).

### SHEDs regulate the secretion of immunoglobulin in an AR mouse model

**Fig. 3:**
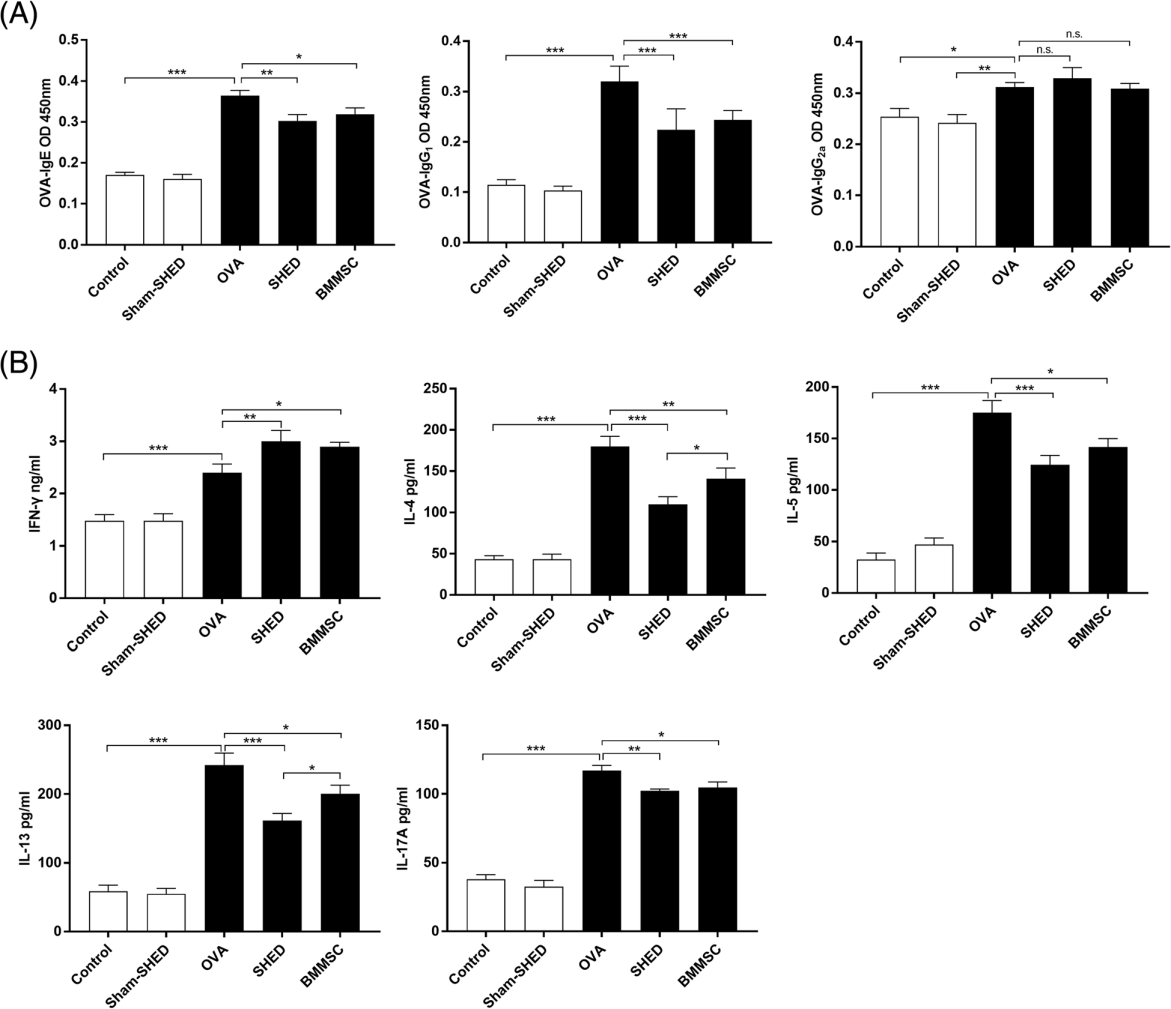
Effect of SHED on antigen-specific antibody and cytokine production. OVA-specific IgE, IgG_1_, and IgG_2a_ levels in serum (**a**) and characteristic cytokines of Th1, Th2, and Th17 cells in lymphocyte culture supernatant (**b**) were measured by ELISA kits. OVA-specific IgE, IgG_1_, and IgG_2a_ levels were higher in the OVA group than in the control and sham-SHED groups. In addition, IgE and IgG_1_ were significantly reduced upon SHED and BMMSC treatments but not IgG_2a_. Both SHED and BMMSC treatments significantly enhanced the expression of IFN-γ but reduced IL-4, IL-5, IL-13, and IL-17A levels. The downregulation of IL-4 and IL-13 levels in the SHED group was more obvious compared with that in the BMMSC group at the same dose. Data are expressed as the mean ± SD (*n* = 6 in each group) from three representative experiments. *p < .05, **p < .01, ***p < .001

Since immunoglobulins play an important role in regulating the inflammatory response, the expression of several OVA-specific immunoglobulin antibodies was determined. As illustrated in Fig. 3a, we confirmed a significant elevation in the serum levels of OVA-specific IgE, IgG_1_, and IgG_2a_ in the OVA group compared with the control and sham-SHED groups (*p* < .05). Interestingly, systemic administrations of SHEDs and BMMSCs resulted in a decrease in serum OVA-specific IgE and IgG_1_ in comparison with the OVA group (SHED: *p* = .002 and *p* < .001; BMMSC: *p* = .018 and *p* < .001) but not IgG_2a_ (SHED: *p* = .419; BMMSC: *p* = .901). The downregulation of IgE and IgG_1_ in the SHED group was not different from that in the BMMSC group at the same injection dose (*p* = .356 and *p* = .188, respectively).

### SHEDs change the cytokine levels in the splenic lymphocyte culture system

To determine whether injected SHEDs affect cytokine production, a lymphocyte restimulation test was performed (Fig. 3b). IFN-γ, IL-4, IL-5, IL-13, and IL-17A levels in the supernatant of OVA-incubated splenocytes were significantly higher in the OVA group than in the control group and sham-SHED group (*p* < .001). IL-4, IL-5 IL-13 and IL-17A levels were significantly reduced upon SHED and BMMSC administration (SHED: *p* < .001, *p* < .001, *p* < .001, *p* = .008, respectively; BMMSC: *p* = .008, *p* = .012, *p* = .002, *p* = .023, respectively). In contrast, the levels of IFN-γ in the supernatant were significantly higher in the SHED and BMMSC treatment groups compared with the OVA group (SHED: *p* = .009; BMMSC: *p* = .027). The downregulation of IL-4 and IL-13 in the SHED group was more obvious than that in the BMMSC group at the same dose (*p =* .031 and *p =* .033, respectively).

### Effect of SHEDs on mRNA expression

**Fig. 4:**
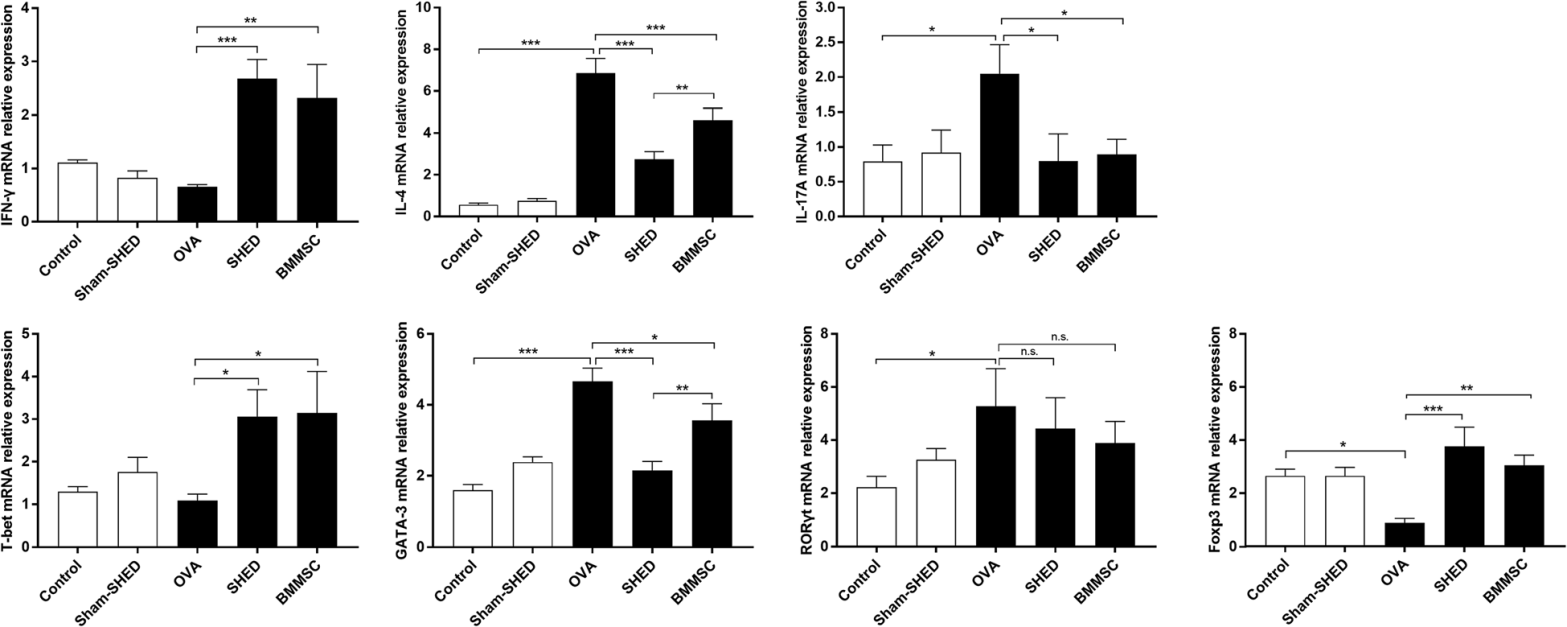
Expression of mRNAs encoding cytokines and specific transcription factors. The relative mRNA expression levels of IL-4 and GATA-3 were significantly higher in the OVA group than in the control and sham-SHED groups, and such upregulation was inhibited by SHED and BMMSC administration. The IFN-γ, T-bet, and Foxp3 mRNA expression levels were markedly enhanced by SHED and BMMSC administration. In addition, MSCs reduced the mRNA expression of IL-17A, but RORγt expression in the SHED and BMMSC treatment groups had no significant change compared with that in the OVA group. The capacity of SHEDs in reducing IL-4 and GATA-3 levels was superior to that of BMMSCs. Data are expressed as the mean ± SD (*n* = 6 in each group) from three representative experiments. *p < .05, **p < .01, ***p < .001, n.s. no significance

To further evaluate the effects of SHED administration on allergic inflammation, we measured the relative expression of mRNAs encoding Th1, Th2, Th17, and Treg cell-related cytokines and specific transcription factors using real-time PCR (Fig. 4). The relative mRNA expression levels of IL-4 and GATA-3 were significantly higher in the OVA group than in the control group and sham-SHED group and were downregulated upon treatment with MSCs (SHED: *p* < .001 for both; BMMSC: *p* < .001 and *p =* .018). The levels of mRNAs encoding IFN-γ and T-bet increased in both the SHED group (*p* < .001 and *p =* .017, respectively) and the BMMSC group (*p =* .002 and *p =* .014, respectively) in contrast to those in the OVA group. Additionally, SHED and BMMSC treatment reduced the mRNA expression of IL-17A in splenic lymphocytes (*p =* .012 and *p =* .02, respectively). However, RORγt expression in the SHED and BMMSC groups showed no significant change compared with that in the OVA group (SHED: *p =* .53; BMMSC: *p =* .303). Furthermore, the relative mRNA expression levels of Foxp3 were significantly decreased in the OVA group compared to the control group (*p =* .006) and were upregulated with the treatment of SHEDs (*p* < .001) and BMMSCs (*p =* .001). The abilities of SHEDs in reducing IL-4 and GATA-3 were stronger than those of BMMSCs (*p =* .006 and *p =* .003, respectively).

### SHEDs inhibit T lymphocyte proliferation in AR patients

**Fig. 5:**
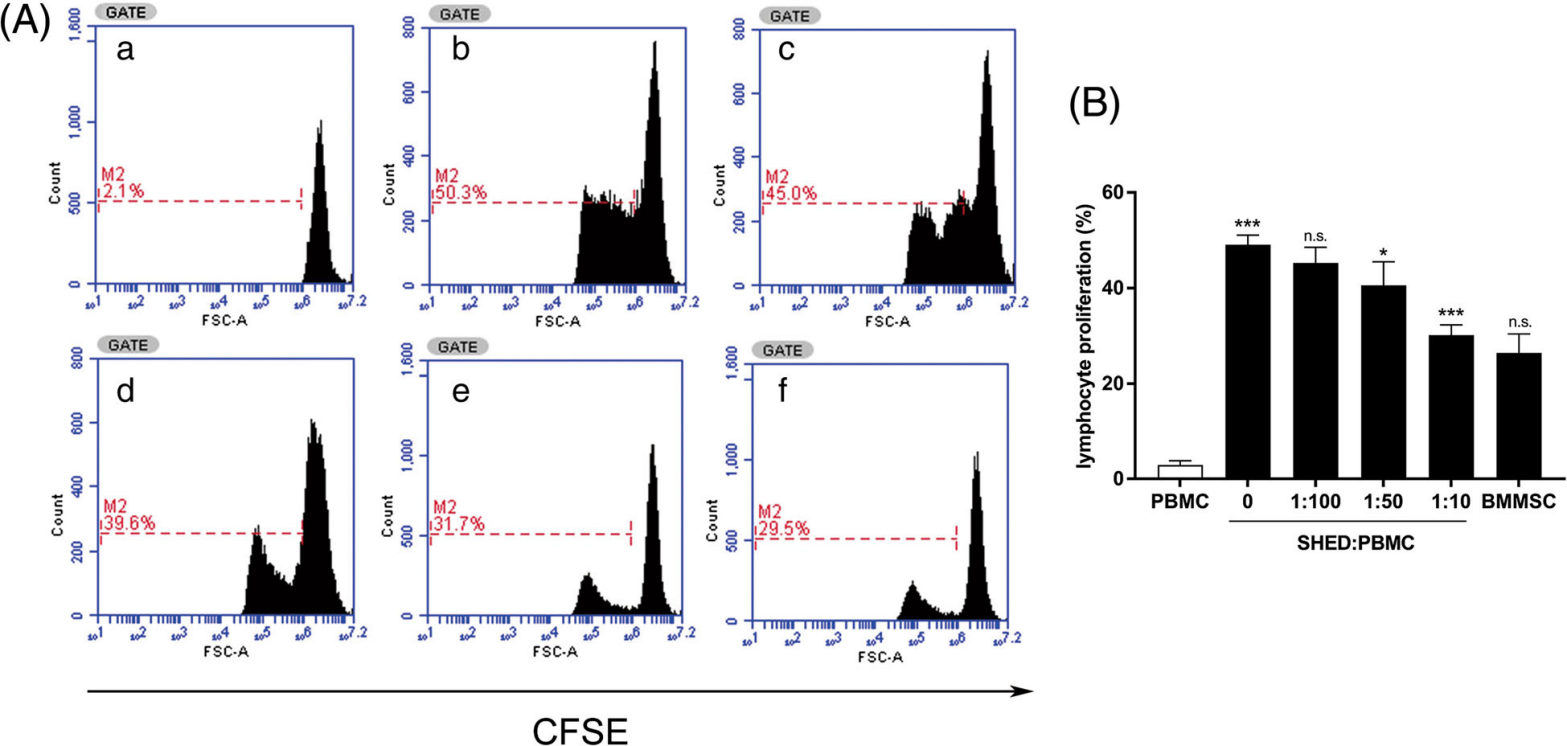
The immunosuppressive effect of SHEDs on PHA-stimulated lymphocyte proliferation. **A** CFSE-labeled PBMCs from AR patients (1 × 10^6^/well) were stimulated by PHA (5 μg/ml) in a 24-well plate for 3 days in the presence or absence of different numbers of SHEDs or BMMSCs (*n* = 5). PBMCs were gated for flow cytometry analysis of CD3^+^ staining. **B** Representative results of T cell proliferation without stimulation (a), PHA (5 μg/ml) stimulation (b), and PHA stimulation in the presence of SHEDs (1 × 10^4^/well, c), SHEDs (2 × 10^4^/well, d), SHEDs (1 × 10^5^/well, d), or BMMSCs (1 × 10^5^/well, e) are shown. SHEDs significantly inhibited PHA-stimulated lymphocyte proliferation in a number-dependent manner, as determined by lymphocyte proliferation percentages. BMMSCs also inhibited T lymphocyte proliferation, but there was no significant difference with SHED at the same dose. The data are expressed as the mean ± SD. *p < .05, ***p < .001, n.s. no significance, compared with group on the left

To explore the immunomodulatory effect of SHEDs on T lymphocytes, a lymphocyte proliferation assay was performed in the CFSE experiment. As illustrated in Fig. 5, we found that the percentages of T lymphocyte proliferation decreased with an increasing SHED to PBMC ratio, suggesting that SHEDs significantly inhibited the proliferation of CD3^+^ T cells in a dose-dependent manner. BMMSCs inhibited PHA-stimulated T cell proliferation as well, but there was no significant difference in inhibition ability between SHED and BMMSC under the same culture conditions.

### SHEDs alter cytokine production in PHA-stimulated PBMCs of AR patients

**Fig. 6:**
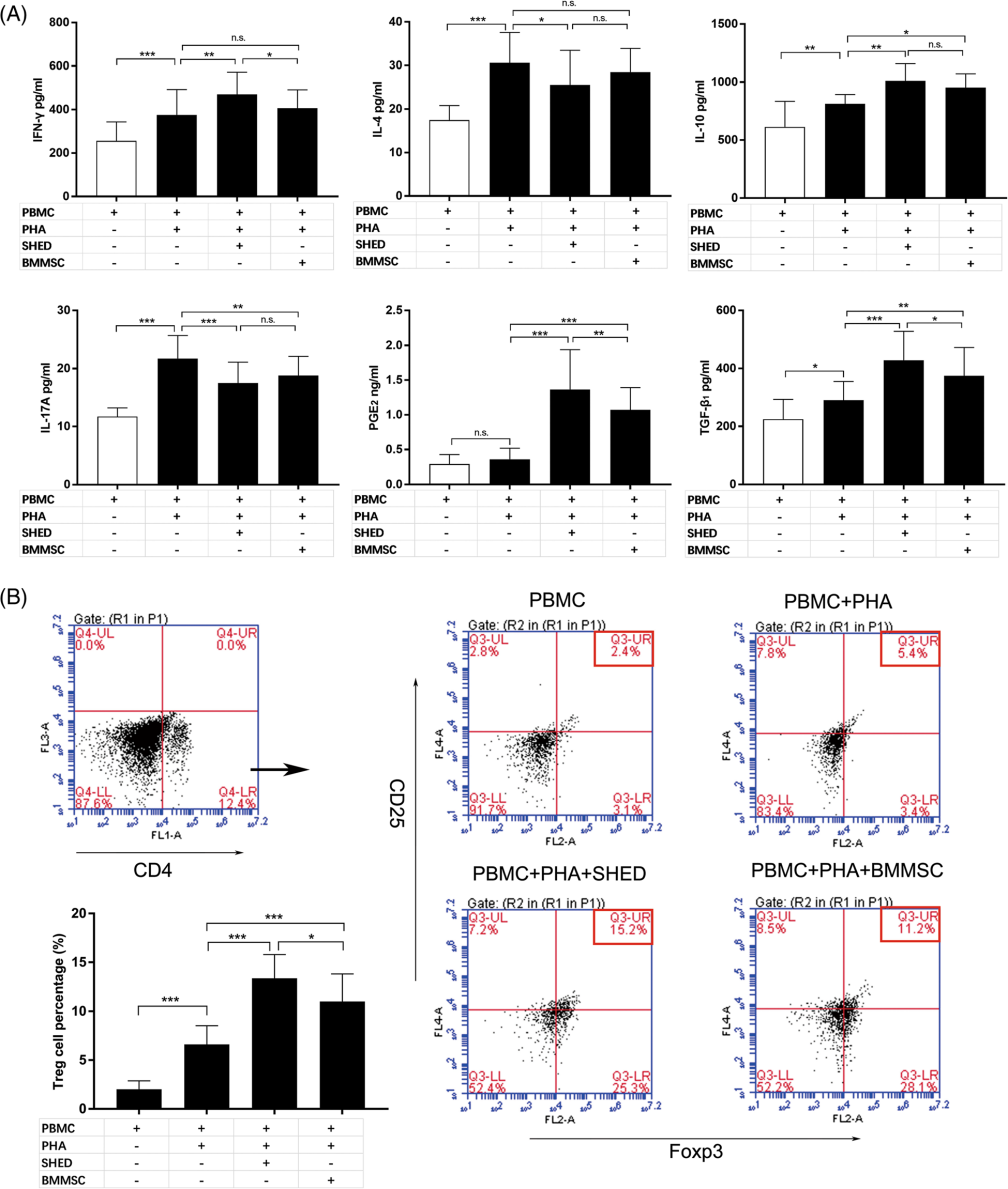
SHED corrected the immune imbalance and promoted the expansion of CD4^+^CD25^+^Foxp3^+^Treg cells in vitro. **a** The addition of SHEDs led to the downregulation of IL-4 and IL-17 levels and the upregulation of IFN-γ, IL-10, PGE_2_, and TGF-β_1_ levels. Although BMMSCs showed similar immunomodulation ability, the levels of IFN-γ and IL-4 were not significantly different from those of the PHA-stimulated PBMC (*n* = 10). **b** In addition, both SHEDs and BMMSCs upregulated the proportion of CD4^+^CD25^+^Foxp3^+^Treg cells in CD4^+^ subsets compared to the PHA-stimulated PBMC group, and the ratio of Treg cells in the SHED group was significantly higher than that in the BMMSC group (*n* = 5). The data are expressed as the mean ± SD. *p < .05, **p < 0.01, ***p < .001, n.s. no significance

To further investigate the immunomodulatory effects of SHEDs on the T cell phenotype, we next examined the effects of SHEDs and BMMSCs on cytokine production in the supernatant using ELISA kits (Fig. 6a). Compared with the culture of PHA-stimulated PBMC, the levels of IFN-γ and IL-10 were significantly increased (*p* = .003, *p* = .006, respectively), and the levels of IL-4 and IL-17A were decreased upon the presence of SHEDs (*p* = .012 and *p* < .001, respectively). Although IL-10 was increased and IL-17A was markedly decreased after the addition of BMMSCs (*p* = .047 and *p* = .006, respectively), there was no significant difference in the levels of IFN-γ and IL-4 between the BMMSC coculture system and the PHA-stimulated PBMC (*p* = .326 and *p* = .294, respectively).

Moreover, MSCs could regulate immune response by secreting soluble factors, such as PGE_2_ and TGF-β_1_. In our results, the levels of PGE_2_ and TGF-β_1_ were increased in the supernatant of SHED or BMMSC coculture system compared to those in the culture of PHA-stimulated PBMC without MSCs (PGE_2_, both *p* < .001; TGF-β_1_, *p* < .001 and *p* = .002, respectively). Significant differences were detected in the levels of PGE_2_ and TGF-β_1_ between the cultures of SHED and BMMSC (*p* = .009 and *p* = .045, respectively).

### SHEDs enhance CD4^+^CD25^+^Fxop3^+^ Treg cell expansion

The effects of SHEDs and BMMSCs on Treg expansion in restimulated PBMCs were determined using flow cytometry. As illustrated in Fig. 6b, we found that both SHEDs and BMMSCs significantly increased the ratio of CD4^+^CD25^+^Foxp3^+^ Tregs in the CD4^+^ subpopulation (*p* < .001). Interestingly, the increase in Foxp3^+^Treg frequency in the SHED group was higher than that in the BMMSC group (*p* = .026).

### TGF-β_1_ neutralization downregulates Treg cells in AR patients

**Fig. 7:**
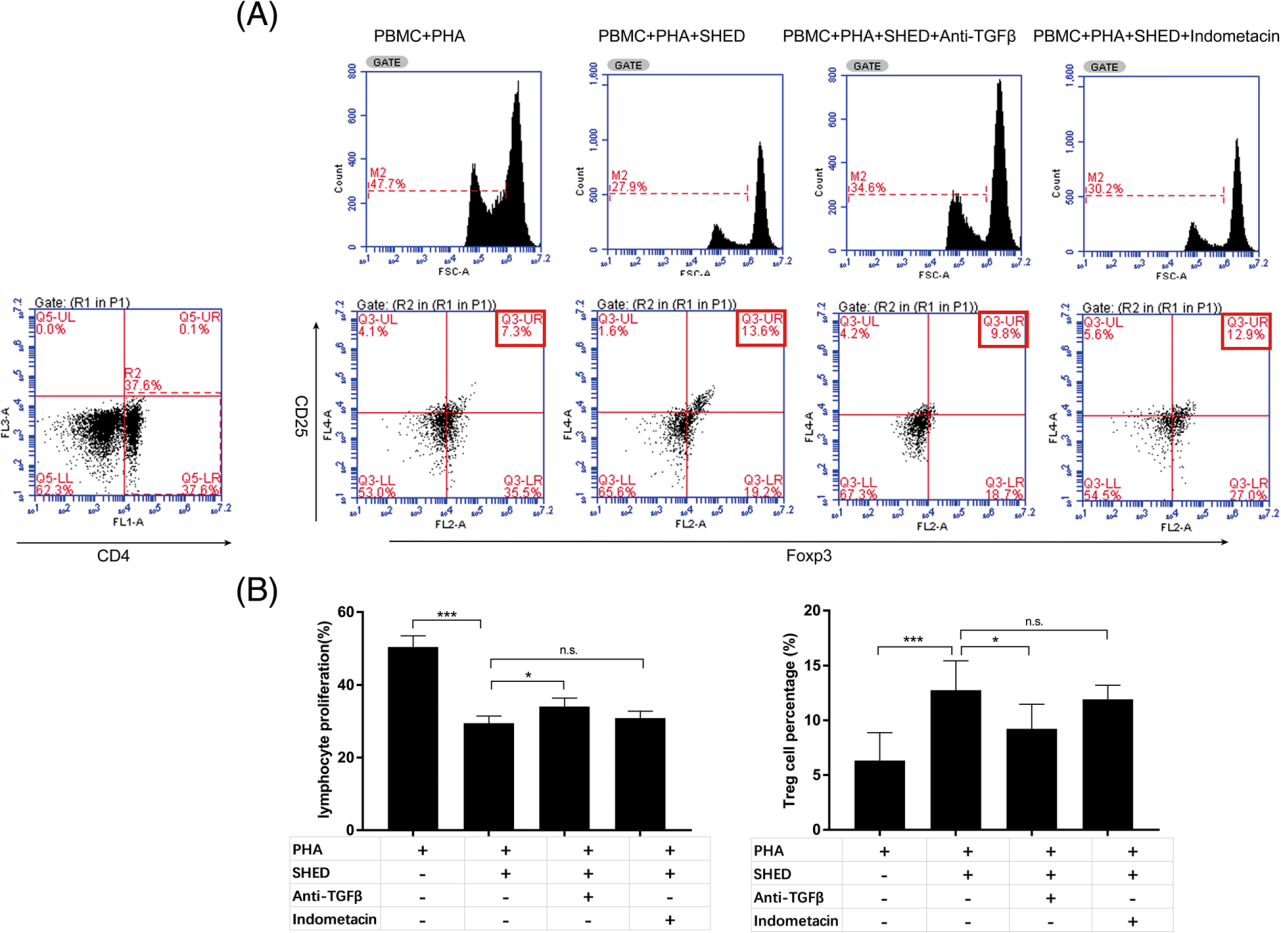
Results of the cytokine neutralization assay. **a** To reveal the role of TGF-β_1_ and PGE_2_ in the immunomodulatory mechanism of SHEDs, a cytokine neutralization assay was performed. Treg expansion was detected via flow cytometry, and T lymphocyte proliferation was evaluated by CFSE labeling (*n* = 5). **b** The percentage of T lymphocyte proliferation was significantly upregulated compared to that in the PBMC+PHA+SHED group upon anti-TGF-β Ab addition, and the percentage of Treg cells markedly decreased. In contrast, there was no significant change in the percentage of T lymphocyte proliferation and Treg cell frequency when indomethacin was added to block the PGE_2_ pathway. The data are expressed as the mean ± SD. *p < .05, ***p < .001, n.s. no significance

As illustrated in Fig. 7, after neutralizing TGF-β_1_, the percentage of Tregs in flow cytometry was significantly decreased in contrast to the SHED group, and the percentage of MFI in the T lymphocyte proliferation test correspondingly increased (*p* = .014 and *p* = .046, respectively). However, these changes were not significant after blocking PGE_2_ (*p* = .531 and *p* = .484, respectively), which suggested that TGF-β_1_ neutralization, but not PGE2 blockage, could partly relieve the immunomodulatory effect of SHEDs on T lymphocytes though changing Treg expansion.

## Discussion

MSCs have been revealed to possess immunomodulatory capacity in vitro and vivo, which significantly alleviates the inflammatory response in allergic diseases [15]. Due to the low expression of major histocompatibility complex class I (MHC I) and the negative expression of major histocompatibility complex class II (MHC II) [16], we transplanted xenogeneic SHEDs into an AR mouse model to explore the immunomodulatory effect compared with that of BMMSCs. In addition, coculture systems of MSCs and PBMCs from AR patients were established to determine the underlying mechanism.

The immunological basis of AR pathogenesis is the decrease of Th1/Th2 ratio. Hence, correcting the imbalance is the key to AR treatment [17]. In the present study, we demonstrated that xenogeneic MSCs downregulated the Th2 inflammatory response and relieved nasal symptoms and inflammation (eosinophil infiltration, goblet cell hyperplasia, and T lymphocyte infiltration). Both SHEDs and BMMSCs significantly reduced the levels of IL-4, IL-5, and IL-13 (representing the Th2 phenotype) in splenic lymphocytes and decreased the production of IgE and IgG_1_ in serum. The relative mRNA expression of IL-4 and GATA-3 was reduced upon MSC injection, which reflected the immunosuppression effect from another aspect. In contrast, SHEDs and BMMSCs upregulated the level of IFN-γ and the relative mRNA expression of IFN-γ and T-bet (representing the Th1 phenotype), suggesting that MSCs could shift the Th2 to Th1 immune response and increase the ratio of Th1/Th2. The above data were consistent with previous studies involving BMMSCs and adipose mesenchymal stem cells (ASCs) [5, 7, 18].

Th17 cells involved in neutrophil and macrophage infiltration are closely related to the initiation and progression of AR [19]. Yamaza et al. [20] found that SHEDs had significant effects on inhibiting Th17 cells in vitro. A recent study also showed that ASCs reduced the IL-17 levels of bronchoalveolar lavage fluid (BALF) and serum in OVA-induced asthma models and suppressed the relative mRNA expression of IL-17 and RORγt in lung tissue [21]. According to our results, although the mRNA expression of RORγt did not change, the levels of IL-17A and mRNA expression of IL-17A in the spleen decreased significantly after MSC transplantation, suggesting that both SHEDs and BMMSCs inhibited the activity of Th17 cells in AR mice and delayed the development of disease.

Moreover, Treg cells characterized by intracellular expression of the transcription factor Foxp3 have been identified as having suppressor functions, which are essential for the resolution of inflammatory processes [15, 22]. Numerous studies have shown the upregulation of Treg cell numbers and activity after the administration of MSCs in asthma models [23, 24]. Similarly, our in vivo study found that Foxp3 mRNA expression in splenic lymphocytes significantly increased in MSC groups compared to the OVA group, providing evidence that SHEDs and BMMSCs play anti-inflammatory roles through Foxp3^+^ Treg expansion in mouse models of AR.

It was acknowledged that MSCs suppressed the immune response by inhibiting T cell proliferation, whether in a mitogen- or allergen-stimulated culture system [25, 26]. To further explore the ability of inhibiting T cell proliferation in vitro, SHEDs and BMMSCs were cocultured in PBMCs from AR patients respectively. Likewise, in our study, we found that SHEDs inhibited CD3^+^ T cell proliferation by decreasing the division percentage in a dose-dependent manner compared to that of PHA-stimulated PBMCs alone. In addition, the decrease in IL-4 and IL-17A and the increase in IFN-γ in the supernatant suggested that SHEDs could increase Th1/Th2 ratio and correct the immune imbalance of AR in vitro. Some studies showed that the level of IFN-γ was decreased after coculture of MSCs with PBMCs from healthy subjects, which was contrary to our results [27, 28]. As the immunosuppressive properties of MSCs were not inflexible but were induced by environmental inflammatory mediators [29], we attribute these contrasting results to the different modulatory effects of MSCs on Th1- or Th2-mediated responses.

The protection afforded by MSCs in allergic disease is mediated through multiple mechanisms (soluble factors, cell-to-cell contact, regulatory T cells, induction of anergy, apoptosis, etc.), which in the end lead to the induction or expansion of active Tregs and the generation of tolerance [30–32]. Furthermore, the promotion of Tregs by MSCs in vitro required cell-cell contact as well as PGE_2_ and TGF-β_1_. Cell contact between MSCs and activated T cells induces IL-10 production, which plays an essential role in promoting the generation of Treg cells [33, 34]. The soluble factors TGF-β_1_ and PGE_2_ are regarded as the main inducers of Treg cell subsets during the differentiation process of T helper lymphocytes [35]. Cho et al. showed that blocking PGE_2_ and neutralizing TGF-β_1_ eliminated the immunosuppressive effect of ASCs [24]. Genç et al. stated that human dental pulp MSCs enhanced CD4^+^CD25^+^Foxp3^+^ Treg frequency and demonstrated that blockade of TGF-β_1_ resulted in decreased T regulatory cell frequency [28]. In accordance with previous studies, Treg cell frequencies were increased in both SHED and BMMSC coculture systems, accompanied by the upregulation of IL-10, TGF-β_1_, and PGE_2_ levels. In addition, neutralizing TGF-β_1_ downregulated Treg cell frequency in contrast to SHEDs cocultured without blockade, leading to the partial relief of the proliferation inhibition of CD3^+^ T cells, which suggested that the change in TGF-β_1_ might be the potential pathway of SHEDs promoting Treg expansion. However, blocking of PGE_2_ did not significantly change the frequency of Treg cells and T lymphocyte proliferation, which indicated that the suppressive mechanism of SHEDs might not be mediated through PGE_2_. Even so, we cannot completely deny the role of PGE_2_ in SHED immunomodulation. MSC-mediated immunoregulation is a redundant system that is mediated by several molecules, and inhibition of any one of these molecules does not result in a complete loss of the immunosuppressive activity of MSCs.

In this study, we compared the immunomodulatory function of SHEDs and BMMSCs in parallel. In vivo, SHEDs were superior to BMMSCs in inhibiting the Th2 immune response (downregulation of IL-4 and IL-13 and mRNA expression of IL-4 and GATA-3) and inflammatory infiltration of nasal mucosa. In vitro, although there was no difference between SHEDs and BMMSCs in the inhibition of T lymphocyte proliferation, the ability of SHEDs to induce the expansion of Treg cells was superior to that of BMMSCs. Moreover, we found an interesting phenomenon in our research. In vitro, the cytokine levels were all significantly changed in SHED coculture system. However, in BMMSC coculture system, even the levels of IFN-γ and IL-4 showed a change tendency, there was no statistical significance compared with the positive control group. This results suggested that SHEDs could achieve the effect of cytokine regulation under the present condition, while BMMSCs might need a larger proportion of cells than 1:10 (MSC to PBMC) or a lower cell passage. According to the above analysis and considering the convenience of SHED acquisition, no ethical issues, and the ability of proliferation, we believe that SHEDs may have more advantages in the application of AR therapy than BMMSCs.

We acknowledge the limitations of this study. For example, it may be more persuasive to investigate the immunoregulation effect of MSCs in mouse splenic lymphocytes directly. However, we chose to use PBMCs from AR patients for the aim of clinical transformation. Second, a transwell assay is suggested to be used to detect the role of cell-to-cell contact on the immunoregulatory capacity of SHED. At last, a coculture assay of higher ratios of MSC to PBMC or lower cell passages need to be performed in the follow-up study.

## Conclusions

SHEDs reduced nasal inflammation, suppressed the Th2-driven response, and corrected the CD4^+^ T cell immune imbalance in a mouse model of AR. In vitro, SHEDs inhibited the proliferation of T lymphocytes and increased the ratio of Th1/Th2 by inducing the expansion of Treg cells. Furthermore, the neutralization of TGF-β_1_ inhibited the immunomodulatory effect of SHEDs, suggesting that TGF-β_1_ plays a crucial role in the underlying mechanism. These results suggest that SHEDs have excellent immunomodulation ability and may be potential therapeutic agents for the treatment of allergic diseases such as AR in the future.

## Additional file

Additional file 1: **Table** **S1.** Corresponding primers designed for quantitative real-time PCR in this study. (DOC 35 kb)

## Abbreviations

APC: Allophycocyanin
AR: Allergic rhinitis
ARIA: Allergic Rhinitis and its Impact on Asthma
ASC: Adipose mesenchymal stem cell
BALF: Bronchoalveolar lavage fluid
BMMSC: Bone marrow mesenchymal stem cell
CFSE: Carboxyfluorescein diacetate succinimidyl ester
COX: Cyclooxygenase
DMEM/F12: Dulbecco’s modified Eagle’s medium/nutrient f-12 ham
FBS: Fetal bovine serum
FITC: Fluorescein isothiocyanate
IFN: Interferon
IHC: Immunohistochemistry
IL: Interleukin
MFI: Mean fluorescent intensity
MHC I: Major histocompatibility complex class I
MHC II: Major histocompatibility complex class II
mRNA: Messenger RNA
MSC: Mesenchymal stem cell
OVA: Ovalbumin
PAS: Periodic acid-Schiff
PBMC: Peripheral blood mononuclear cell
PE: Phycoerythrin
PGE_2_: Prostaglandin-E2
PHA: Phytohemagglutinin
SHED: Stem cell from human exfoliated deciduous teeth
TGF-β_1_: Transforming growth factor beta 1
α-MEM: Alpha modification of Eagle’s medium
