# Supplementary material for: Stem cells from human exfoliated deciduous teeth correct the immune imbalance of allergic rhinitis via Treg cells in vivo and in vitro
Source: Stem Cell Res Ther. 2019 Jan 22;10:39. doi: 10.1186/s13287-019-1134-z (PMC6341645; doi:10.1186/s13287-019-1134-z)
Supplement: Supplementary file 1 — Table S1. Corresponding primers designed for quantitative real-time PCR in this study. (DOC 35 kb) [file 13287_2019_1134_MOESM1_ESM.doc]

**Table S1. Corresponding primers designed for quantitative real-time PCR in this study**

| **Primer name** | **Primer sequences ( 5′-to-3′direction)** | **Size (bp)** |
| --- | --- | --- |
| IFN-γ forward primer | TAACTCAAGTGGCATAGATGTGGAAG | 169 |
| IFN-γ reverse primer | GACGCTTATGTTGTTGCTGATGG |
| IL-4 forward primer | TCGTCTGTAGGGCTTCCAAGGTGCT | 166 |
| IL-4 reverse primer | GTGGACTTGGACTCATTCATGGTGC |
| T-bet forward primer | GCAAGGACGGCGAATGTT | 133 |
| T-bet reverse primer | CACCCACTTGCCGCTCTG |
| GATA-3 forward primer | CTGGAGGAGGAACGCTAATGG | 131 |
| GATA-3 reverse primer | TGCTAGACATCTTCCGGTTTCG |
| IL-17A forward primer | CCTCAGACTACCTCAACCGTTCC | 191 |
| IL-17A reverse primer | AGGCTCCCTCTTCAGGACCAG |
| RORγt forward primer | GGTCCAGACAGCCACTGCATTC | 153 |
| RORγt reverse primer | GGTGCGCTGCCGTAGAAGGT |
| Foxp3 forward primer | CTTCAAGTACCACAATATGCGACCC | 256 |
| Foxp3 reverse primer | CTTGCGAAACTCAAATTCATCTACGGT |
| β-actin forward primer | CGTTGACATCCGTAAAGACCTC | 159 |
| β-actin reverse primer | ACAGAGTACTTGCGCTCAGGAG |

The messenger RNA (mRNA) [relative expression](../../../../C:/Program%20Files%20(x86)/Youdao/Dict/7.5.2.0/resultui/dict/javascript:%3B) analysis of IFN-γ, T-bet, IL-4, GATA-3, IL-17A, RORγt and Foxp3. The average transcript levels of genes were normalized to β-actin.
